# Study of an *Enterococcus faecium* strain isolated from an artisanal Mexican cheese, whole-genome sequencing, comparative genomics, and bacteriocin expression

**DOI:** 10.1007/s10482-024-01938-0

**Published:** 2024-02-23

**Authors:** Daniel Acero-Pimentel, Diana I. Romero-Sánchez, Sac Nicté Fuentes-Curiel, Maricarmen Quirasco

**Affiliations:** Departamento de Alimentos y Biotecnología, Facultad de Química, Universidad Nacional Autónoma de México, Ciudad Universitaria, 04510 Mexico City, Mexico

**Keywords:** Antibiotic resistance, Bacteriocins, Comparative genomics, Dairy products, *Enterococcus faecium*, RT-qPCR

## Abstract

Enterococci are ubiquitous microorganisms in almost all environments, from the soil we step on to the food we eat. They are frequently found in naturally fermented foods, contributing to ripening through protein, lipid, and sugar metabolism. On the other hand, these organisms are also leading the current antibiotic resistance crisis. In this study, we performed whole-genome sequencing and comparative genomics of an *Enterococcus faecium* strain isolated from an artisanal Mexican Cotija cheese, namely QD-2. We found clear genomic differences between commensal and pathogenic strains, particularly in their carbohydrate metabolic pathways, resistance to vancomycin and other antibiotics, bacteriocin production, and bacteriophage and CRISPR content. Furthermore, a bacteriocin transcription analysis performed by RT-qPCR revealed that, at the end of the log phase, besides enterocins A and X, two putative bacteriocins not reported previously are also transcribed as a bicistronic operon in *E. faecium* QD-2, and are expressed 1.5 times higher than enterocin A when cultured in MRS broth.

**Supplementary Information:**

The online version contains supplementary material available at 10.1007/s10482-024-01938-0.

## Introduction

Members of the *Enterococcus* genus are non-motile, non-sporulated, Gram-positive lactic acid bacteria (LAB) distributed ubiquitously in many environments. They commonly live in water, soil, animal gut microbiome, and even human milk; they also belong to dairy farm environments such as feed, bedding, and udder and teet surfaces. Consequently, these microorganisms can also be found in food derived from the above sources, e.g., fermented meats and vegetables and dairy derivatives such as cheese (Ben Braïek and Smaoui 2019). It is estimated that enterococci can make up to 1% of the total microbiome of handmade artisanal cheeses, similar to the human fecal microbiota composition (Dubin and Pamer 2017; Escobar-Zepeda et al. 2016).

Enterococci, however, are also opportunistic pathogens in certain niches, namely hospital environments, because some strains contain a variety of virulence factors and are intrinsically resistant to many common antibiotics such as β-lactams, aminoglycosides, and macrolides (Freitas et al. 2021). Particularly, certain *Enterococcus faecium* strains are resistant to vancomycin (vancomycin-resistant enterococci, VRE)—a phenotype commonly associated with inpatient enterococcal infections—and are of concern worldwide, being at the top of the WHO list of priority pathogens. Currently, VRE are a major target in the development of new antibiotics (World Health Organization 2017). Also, enterococci are a natural reservoir of multiple virulence factors and antibiotic-resistance genes. Unlike most LAB, the genus is neither considered Generally Regarded As Safe (GRAS) nor does it have the Qualified Presumption of Safety (QPS) status, and its role as a probiotic or starter culture is still debated (Dapkevicius et al. 2021).

Virulence factors have distinct roles during the infection process. While some elements in *E. faecium* pathogenic strains may facilitate host invasion (cytolysin, hyaluronidase, gelatinase), other factors participate only in the colonization stage (i.e., aggregation substance, collagen-binding protein, surface protein) (Chajęcka-Wierzchowska et al. 2017). Both pathogenic and commensal enterococcal strains require colonization factors, but only the pathogenic ones contain the invasion elements needed to harm the host (Dapkevicius et al. 2021). Even if commensal strains contain genes related to antibiotic resistance, these are useless because commensal strains lack the invasiveness needed to spread within the host (Beceiro et al. 2013).

In artisanal cheeses made from raw milk, *E. faecium* plays a role in the ripening process via the synthesis of various substances related to the development of textures, odours, and flavours. It also produces various antimicrobial substances that contribute to the innocuity of cheese and preserves it with no need for processes such as pasteurization (Olvera-García et al. 2018). Additionally, commensal strains possess certain genotypical and phenotypical characteristics that allow them to act as probiotics through bowel colonization, which depends on factors such as the aggregation substance and the surface protein (Chajęcka-Wierzchowska et al. 2017). Probiotics may benefit the host by enhancing nutrient and drug bioavailability, regulating the immune system, and boosting the neurological system (Maldonado Galdeano et al. 2019). It is clear that the behaviour of commensal strains differs substantially from the one of their pathogenic relatives.

Given the considerable amount of genotypical and phenotypical variations within the same species, genome-wide studies are an effective method to determine which elements make a strain pathogenic or commensal. In these studies, researchers look for genetic features related to invasion, colonization, antibiotic resistance, immune system evasion, toxin production, biomolecule uptake and usage, flavour and odour production, and bacteriocin synthesis, among others (Apostolakos et al. 2023). The present study explored the genome of *E. faecium* QD-2 strain isolated from an artisanal Cotija cheese prepared locally in the Jalisco-Michoacán region of Mexico via genome-wide studies and RT-qPCR of bacteriocin transcripts.

## Materials and methods

### Strain isolation and DNA extraction

LAB isolation was performed from seven “Region of Origin” artisanal Cotija cheeses. After 10 g of each cheese was homogenized using a Stomacher 400 Circulator (Seward Laboratory, London, UK), with 90 mL of peptone water (0.85% NaCl, w/v; casein peptone 1%, w/v), an enrichment step in Man, Rogosa & Sharpe broth (MRS, Oxoid, Basingstoke, UK) was carried out (37 °C, 250 rpm, 24 h). An aliquot of this culture was inoculated in the selective EVA (Ethyl-Violet-Azide) medium (Condalab, Madrid, Spain) (37 °C, 250 rpm, 48 h) and later streaked in Kanamycin-Aesculine-sodium Azide (KAA, Oxoid) agar. Thirty-nine colonies with the characteristic *Enterococcus* phenotype were selected (kanamycin resistance and black halos due to the hydrolysis of aesculine). Out of these, the 12 isolates which showed a 1090 base pairs (bp) amplicon corresponding the *ddl* (D-Ala:D-Ala ligase) gene, using the primers reported by Depardieu et al., (2004), corresponded to *Enterococcus faecium*. These strains also showed a catalase ( +) and Gram ( +) phenotype. Finally, the antilisterial activity of all strains was tested by agar diffusion tests, in which strain QD-2 showed the largest zone of inhibition. *E. faecium* QD-2 (CFQ-B-304) was deposited in the Culture Collection of the School of Chemistry (CFQ) at *Universidad Nacional Autónoma de México* (UNAM) (WDCM No. 100). For DNA extraction, QD-2 was subcultured statically in MRS broth (Oxoid) at 37 °C for 48 h. DNA was extracted as described by Sambrook et al. (2012).

### DNA sequencing and assembly

Total DNA was sequenced in an Illumina NextSeq 500 platform at the sequencing unit of the *Unidad Universitaria de Secuenciación Masiva y Bioinformática-UNAM* (Massive Sequencing and Bioinformatics University Unit; UUSMB-IBt-UNAM) in a paired-end format, with a depth of 5,000,000 reads and a read length of 75 bp. Read quality and GC content were assessed using the FastQC software (Andrews 2010). Reads were assembled using Spades v3.13.3 (Prjibelski et al. 2020). Assembly integrity and contamination were estimated using CheckM v1.0.18 in KBase (Parks et al. 2015). The scaffolds were oriented in Mauve v20150226 using *E. faecium* ATCC 8459 (GCA_000336405.1) as a template (Darling et al. 2004). N50 and L50 statistics were calculated using an in-house Perl script. Coding regions were predicted and annotated using Prokka v1.13.3 (Seemann 2014) and Parallel Annotation Pipeline (PAP) v1.0 (Estrada 2023). The dataset generated during the present study is available on GenBank under accession number CP130822.

### Genomic analyses

Five pathogenic (Aus0004, Aus0085, DO, V1164 and V1836; GCA_000250945.1, GCA_000444405.1, GCA_000174395.2, GCA_020162175.1 and GCA_008728455.1, respectively) and four dairy-related *E. faecium* strains (D, ATCC 8459, DRD-156 and IQ110; GCA_002006745.1, GCA_000336405.1, GCA_023743905.1 and GCA_001455445.1, respectively) were selected to carry out the comparative genomic analyses against QD-2. Pangenomic clustering of all ten strains was performed using the Cluster of Orthologous Groups (COG) and OrthoMCL algorithms, and a maximum likelihood tree was generated using GET_HOMOLOGUES v3.5.5 (Contreras-Moreira and Vinuesa 2013). The pangenomic structure was graphically represented using Roary v3.11.2 (Page et al. 2015). The genomic contents of all strains was further analysed using OrthoVenn v3.0, using the OrthoFinder algorithm (Sun et al. 2023).

Virulence factors were surveyed in the Virulence Factor Database (VFDB) (Chen 2004), and antibiotic resistance genes were screened using AMR++ v3.0 (Bonin et al. 2022). Genomes were screened for CRISPR-Cas sequences using CRISPRCasFinder (Couvin et al. 2018), and bacteriophage contents were explored using PHASTER (Arndt et al. 2016). Integron presence was assessed using Integron_Finder v2.0.2 (Néron et al. 2022).

The presence of genes related to sugar metabolism was confirmed using dbCAN3 (Zheng et al. 2023), and genes related to proteolysis, lipolysis, and biogenic amine synthesis were detected using the KEGG Automatic Annotation Server (KAAS) (Moriya et al. 2007).

A prediction of bacteriocin-coding sequences in *E. faecium* QD-2 was carried out using BAGEL4 v1.2 (van Heel et al. 2018), and the resulting sequence identities were confirmed using the blastp suite in NCBI and UNIPROT (Altschul et al. 1990). Sigma 70 promoter sequences were predicted using IPro70-FMWin (Rahman et al. 2018).

### Bacteriocin expression

*E. faecium* QD-2 was cultured and incubated statically in 25 mL MRS broth (Oxoid) at 37 °C for 18 h. Its growth curve was plotted, and the optimal timepoint for RNA extraction was determined as six hours (end of the log phase). RNA was extracted with the TRIzol method (TRI Reagent^®^, Sigma-Aldrich, St Louis, MO) as described by the manufacturer. RNA integrity was assessed by agarose gel electrophoresis (1% agarose–18% formaldehyde, Sigma-Aldrich). The transcripts of interest were amplified via RT-qPCR using the SCRIPT RT-qPCR SybrMaster kit (Jena Bioscience, Dortmund, Germany), following the manufacturer’s instructions. Primers were designed from the predicted bacteriocin-coding open reading frames (ORFs) and synthesised by Integrated DNA Technologies, Inc. (Coralville, IA) (Table 1).

**Table 1:** Oligonucleotides used in the RT-qPCR experiments

ORF / Gene	Nucleotide sequence (5'–3')
615	F: TGGAGGCAATAATGCTTGGG
	R: CTAAACCTGCACCACCTACTG
616	F: GAGTGCCGTGGGAGTTT
	R: CACAAGCAGCTATTGCATAAGG
621 (e*ntA*)	F: AAATAAATGTACGGTCGATTGGG
	R: CCTGGAATTGCTCCACCTAAA
901 (e*nxA*)	F: ATGATAGTCTTTGGTATGGTGTAGG
	R: TCATGTGTTTAACAGGATGGTTTG
902 (e*nxB*)	F: TTCAAGGAGGAATAGCACCTATTAT
	R: AGATTATTTGATCTGAGTGATCCCA
*rpoA*	F: CCTGTTCGTCGTGTGAACTATC
	R: ACCATCTGTCCAAATCTCCATC

The relative expression of the putative bacteriocins of interest (ORFs 615, 616, 901, and 902) against the reference gene (enterocin A, ORF 621) was calculated using the ΔΔCt method. It consists of the normalization of the respective cycle threshold (Ct) values against an endogenous reference gene (in our case, *rpoA*) and the comparison of the normalised expression (ΔCt) against a reference gene of interest (ΔΔCt), expressed as 2^−ΔΔCt^ (Livak and Schmittgen 2001).

### Statistical analysis

RT-qPCR data were subjected to a one-way Analysis of Variance (ANOVA) (α = 0.05) in R v4.2.3 and GraphPad Prism v8. The normality of the dataset was assessed using Q-Q plots and the Shapiro–Wilk test. The data homoscedasticity was determined using Bartlett’s test, and *post-hoc* differences between groups were calculated using Tukey’s range test.

## Results and discussion

The present study was carried out to assess the similarities and differences between a food *Enterococcus faecium* strain (QD-2) isolated from a naturally ripened Mexican cheese (Cotija cheese) against four other dairy-related strains (D, ATCC 8459, DRD-156 and IQ110) and five pathogenic ones (Aus0004, Aus0085, DO, V1164 and V1836). Strain D was also isolated from Cotija cheese (Olvera-García et al. 2018).

### Comparative genomics

Total DNA extraction from *E. faecium* QD-2 revealed the absence of plasmid material; the FastQC read quality evaluation at each nucleotide position was ≥ 26, which is considered adequate. Table 2 shows the statistical analyses of the sequencing procedure. Since integrity was greater than 99% and contamination was 0%, the draft genome was considered suitable for the subsequent bioinformatic analyses.

**Table 2:** Sequencing statistics and genome features of *Enterococcus faecium* QD-2

Feature	Value
Genome size (bp)	2,676,947
Number of scaffolds	97
N50 (bp)	110,598
L50	8
Largest scaffold (bp)	344,463
Shortest scaffold (bp)	211
GC (%)	~ 39
Integrity (%)	99.63
Contamination (%)	0.0
CDSs	2565
rRNAs	3
tRNAs	46
tmRNAs	1

**Fig. 1:**
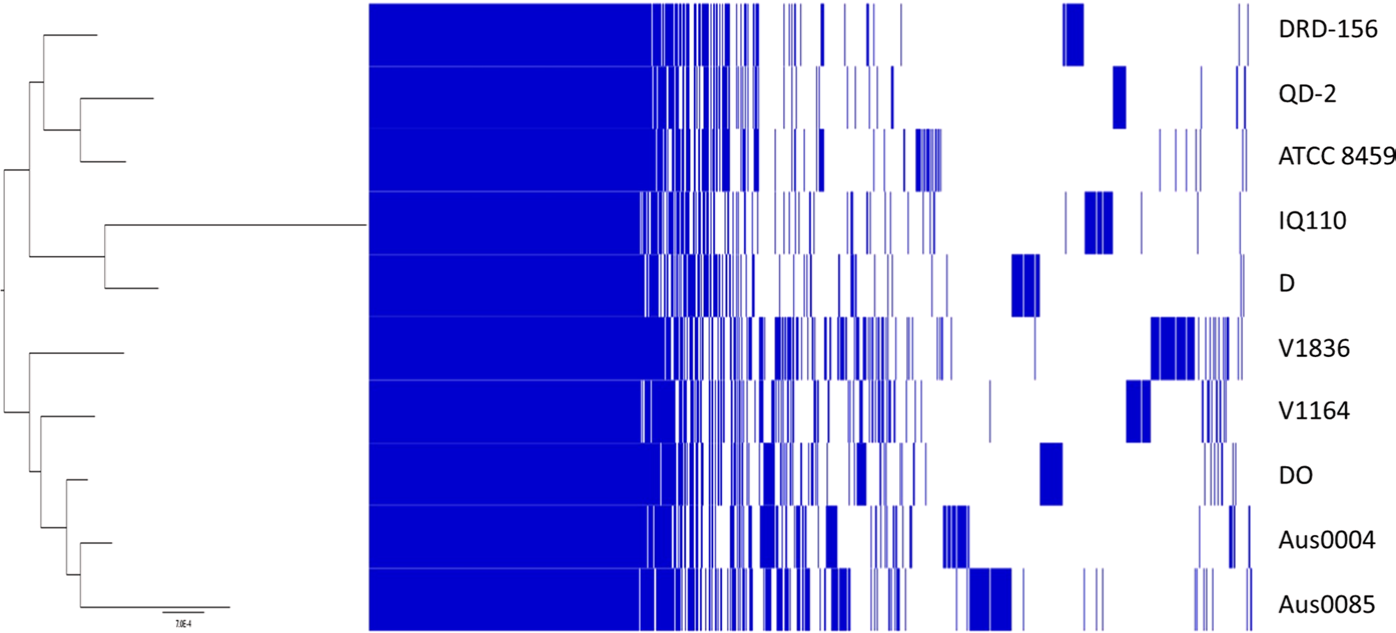
Pangenomic structure and maximum likelihood tree of *Enterococcus faecium* strains: five pathogenic (Aus0004, Aus0085, DO, V1164 and V1836) and five commensal strains (ATCC 8459, D, QD-2, DRD-156 and IQ110)

First, the pangenomic structure of the ten strains was determined, showing a clear division into two distinct clades (pathogenic and commensal) (Fig. 1). The genomic core contains all genetic elements that are essential for living and those that characterise the *Enterococcus* genus. Of particular interest are the features that vary between pathogenic and commensal strains. The genomic plasticity of the species can be visually appreciated by simply comparing the absence or presence of certain genomic elements between strains. These include mobile elements (phage-related), virulence factors, antibiotic resistance, metabolic features, and bacteriocin-coding genes, among others (Fig. 1 and Table 3, 4, 5 and S1). Studies on *E. faecium, E. faecalis*, and *E. hirae* have shown that, even at the species level, virulence-associated elements, such as biofilm formation and cell adhesion, and carbohydrate metabolism are highly niche-dependent (Zaidi et al. 2022). Additionally, food-related enterococcal strains tend to have multiple copies of those genes required to use specific carbohydrates, such as lactose, and of certain proteolytic genes due to the selective pressure of the medium (Olvera-García et al. 2018). Enterococci possess high genomic flexibility, allowing them to acquire various mobile elements associated with pathogenicity and survival capability in a wide range of ecosystems (Boumasmoud et al. 2022). However, the acquisition and usage of these elements are highly dependent on the niche and the conditions in which the microorganism inhabits (Das et al. 2022).

**Table 3:** Bacteriophage elements identified in the *Enterococcus faecium* strains studied

Strain	Phage species	Bacterial genus of origin	Completeness (0–150)	Region size (kb)	Number of proteins
QD-2	NC_004305	*Lactobacillus*	150	40.1	53
	NC_003291	*Listeria*	130	41.7	46
D	NC_028830	*Lactobacillus*	110	39.4	49
	NC_028671	*Enterococcus*	130	44.7	62
ATCC 8459	NC_004305	*Lactobacillus*	150	21.5	25
IQ110	None	*–*	–	–	–
DRD-156	NC_003291	*Listeria*	110	36.9	58
Aus0004	NC_003291	*Listeria*	130	49.3	58
	NC_003291	*Listeria*	150	56.5	66
	NC_003291	*Listeria*	120	38.9	60
Aus0085	NC_021539	*Listeria*	150	62.7	69
	NC_004821	*Bacillus*	110	48.9	62
	NC_013646	*Enterococcus*	150	44.1	63
	NC_028826	*Enterococcus*	150	58.4	62
	NC_028854	*Paenibacillus*	130	36.2	29
	NC_029119	*Staphylococcus*	140	37.0	27
DO	NC_003291	*Listeria*	140	48.7	65
	NC_028837	*Paenibacillus*	130	19.6	23
	NC_002486	*Staphylococcus*	130	33.8	24
V1164	NC_003291	*Listeria*	140	40.5	64
	NC_029119	*Staphylococcus*	110	33.1	22
	NC_029119	*Staphylococcus*	130	30.1	21
V1836	NC_009812	*Listeria*	100	39.2	59
	NC_003291	*Listeria*	150	42.0	72
	NC_029119	*Staphylococcus*	140	30.6	26
	NC_029119	*Staphylococcus*	110	20.7	26
	NC_029119	*Staphylococcus*	130	27.6	41

**Table 4:** Virulence factors detected in VFDB the *Enteroroccus faecium* strains studied

Function	Virulence factors	Genes	QD-2	D	ATCC 8459	IQ110	DRD—156	Aus0004	Aus0085	DO	V1164	V1836
Adherence	Acm	*acm*	X		X			X	X	X	X	X
	Ebp pili	*ebpA*	X	X		X	X	X	X	X	X	X
		*ebpB*	X	X		X	X	X	X	X	X	X
		*ebpC*	X	X		X	X	X	X	X	X	X
		*srtC*	X	X		X	X	X	X	X	X	X
	EcbA	*ecbA*						X	X	X		X
	EfaA	*efaA*	X	X	X	X	X	X	X	X	X	X
	Esp	*esp*						X	X			X
	Scm	*scm*			X		X	X	X	X	X	X
	SgrA	*sgrA*						X	X	X	X	X
Anti-phagocytosis	Capsule	*cpsA/uppS*	X	X	X	X	X	X	X	X	X	X
		*cpsB/cdsA*	X	X	X	X	X	X	X	X	X	X
Biofilm formation	BopD	*bopD*	X	X	X	X	X	X	X	X	X	X
Toxin	Hemolysin	*hylA*								X	X	X
Immune evasion	Capsule	WP_038810135.1	X									
		*eps3*	X									
		*wchJ*	X									
		*epsE*	X							X	X

**Table 5:** Number of antibiotic resistance genes found in the *Enteroroccus faecium* strains studied

Resistance	QD-2	D	ATCC 8459	IQ110	DRD-156	Aus0004	Aus0085	DO	V1164	V1836
Aminoglycosides	1	1	2	1	1	1	3	4	3	4
Betalactams	1	1	1	1	1	1	1	1	1	1
Elfamycins	1	1	1	1	1	1	1	1	1	1
Aminocoumarins	1	1	1	1	1	1	1	1	1	1
Macrolides, Lincosamides, Streptogramins	1	1	1	1	1	3	5	2	2	1
Multi-drug efflux pumps	1	1	1	2	1	1	3	1	1	2
Fluoroquinolones	0	0	1	1	1	0	1	1	2	2
Glycopeptides (Vancomycin)	0	0	0	0	0	5	5	0	5	5
Nucleosides	0	0	0	0	0	0	2	3	1	0
Tetracyclines	0	0	0	0	0	0	1	2	2	1
Trimethoprim	0	0	0	0	0	1	1	0	1	1
Chloramphenicol	0	0	0	0	0	0	0	1	1	0

**Fig. 2:**
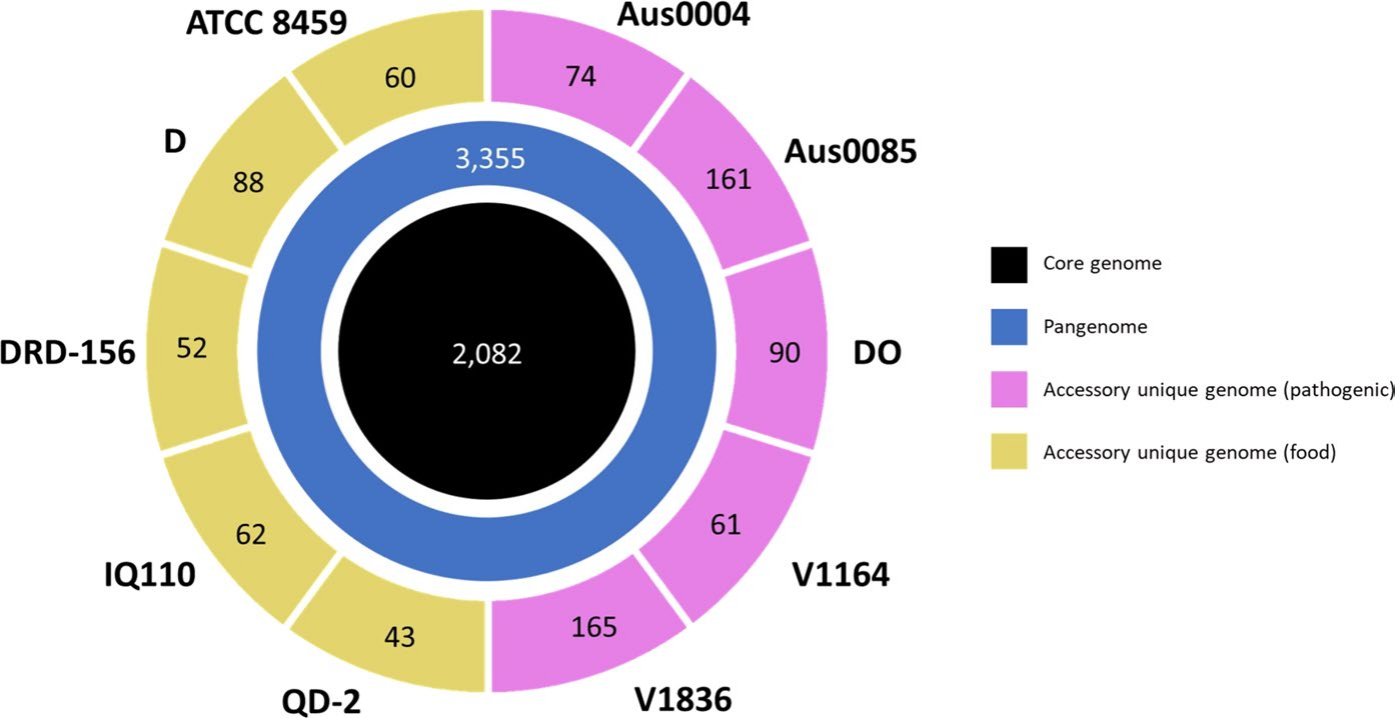
Core-, pan- and unique genome structure of all food (ATCC 8459, D, DRD-156, IQ110 and QD-2) and pathogenic (Aus0004, Aus0085, DO, V1164 and V1836) strains

Venn diagrams are useful for comparing the shared elements between organisms. In the present study, these diagrams reveal that their pangenome comprise 3355 genes, out of which 2082 belong to the core genome. Accessory genes shared between QD-2 and the pathogenic strains comprise around 5.5% of the pangenome, i.e. 184 genes (Fig. 2). A manual parsing of these sequences indicates that most correspond to basic metabolic processes such as carbohydrate intake (40%, 73 genes) and bacteriophage-related proteins (20%, 37 genes). This figure differs from the 462 accessory genes shared exclusively between all pathogenic strains, some of which encode proteins related to antibiotic resistance (*vanWSB, tetR*) and plasmid mobilization (*mobC*, *traE*, and *ardA*), the latter encoding an anti-restriction protein that facilitates the establishment of mobile elements in the microorganism and it is present in multiple copies in the pathogenic strains: up to three in Aus0004, Aus0085, V1164 and V1836. Anti-restriction proteins have been associated with antibiotic resistance dissemination, such as carbapenem resistance in *Klebsiella pneumoniae* because they prevent foreign DNA restriction (Liang et al. 2017). The presence of these DNA mobilization elements in the pathogenic strains is consistent with their genomic structure, as all possess multiple plasmids (between 3 and 9); whereas the commensal strains D and QD-2 have none and strains ATCC 8459 and DRD-156 have only one.

#### Bacteriophage infection remnants

The analysis of the ten genomes using PHASTER revealed multiple regions with bacteriophage identity. These elements were compared in Table 3, which shows only those sequences with a completeness score higher than 100, calculated using the platform as described by Arndt et al. 2016. Bacteriophages can make up to 20% of the total genomic content of bacteria and can provide the metabolic means needed to adapt to a particular niche (Casjens 2003). Our findings show that, compared to the pathogenic strains, commensal *Enterococcus* contain fewer prophage sequences inserted into their genomes, and the predominant ones come from LAB-infecting bacteriophages, such as *Lactobacillus* and *Enterococcus* phages. On the other hand, pathogenic strains contain more *Listeria, Staphylococcus* and *Enterococcus* prophages. This pattern suggests the infection history of those strains and the kind of interactions of certain *E. faecium* strains with their environment. This bacteriophage-mediated horizontal transfer behaviour within and between phyla plays a central role in bacterial evolution, and these interactions may trigger the transfer of certain genetic elements pertaining to pathogenicity and virulence in a specific niche, such as a hospital setting (Casjens and Hendrix 2014; Ikhimiukor et al. 2023). It has been reported that certain Gram-negative-infecting viral families, such as *Inoviridae*, transport genes related to biofilm synthesis, immune evasion, and toxin secretion (Burckhardt et al. 2023). Thus, it is not surprising that specific *Enterococcus* strains may have acquired some of their genetic elements from this cross-genus horizontal gene transfer, which is highly dependent on their environment.

Likewise, this behaviour depicts the receptiveness of pathogenic *E. faecium* strains in regard to exogenous DNA. Bacteria defend themselves from bacteriophage infections by means of systems such as CRISPR-Cas, a bacterial immune system that functions through the insertion of small viral fragments into the bacterial genome and the subsequent recognition and restriction of these sequences in case of a second infection. Therefore, this system plays a major role in the evolution of bacterial genomes (Rath et al. 2015). It was observed that none of the studied strains contains a functional CRISPR-Cas system; all contain CRISPR sequences, but no Cas coding genes inserted in their genomes. It has been described that multidrug-resistant *E. faecium* strains lack a functional CRISPR-Cas system (Alduhaidhawi et al. 2022). This may imply that, in a hospital setting, these strains are subjected to a selective pressure favouring the survival of strains unable to decompose exogenous DNA; consequently, they can easily acquire mobile virulence and resistance genes, which may help them thrive in this environment (Tao et al. 2022). This behaviour has been observed and described in VRE *E. faecium* and *E. faecalis* (Alduhaidhawi et al. 2022). On the other hand, food strains are not subjected to this pressure; as a result, there is no clear preference towards the predominance of either CRISPR-Cas-competent or incompetent organisms (Markusková et al. 2018; Oliveira et al. 2022). This study determined that none of the ten strains contain integrons in their genome, but some contain multiple integrase-coding genes. As already mentioned, bacteriophage sequences in the bacterial genome are associated with virulence (Casjens 2003). Particularly, class-1 integrons are responsible for the spread of antibiotic resistance in most Gram-negative pathogenic bacteria and a few Gram-positive ones (Ghaly et al. 2017).

#### Virulence attributes and antibiotic resistance elements

It should also be noted that both commensal and pathogenic strains share multiple biofilm (*bopD*), capsule (*cpsA, cpsB*), and adhesion (*efaA, acm, ebp*) virulence factor genes according to VFDB, which are necessary for their establishment in the host (Table 4). These properties are desirable in potentially probiotic *Enterococcus* strains, and their presence in the probiotic strain *E. faecalis* Symbioflor1 has been reported (Domann et al. 2007).

Despite sharing multiple virulence factors, none of the commensal strains contains the enterococcal surface protein gene, *esp.* Enterococcal strains containing *esp* usually also carry vancomycin-resistance genes (Kafil and Mobarez 2015). Additionally, a manual parsing of the annotated genomes of the pathogenic strains reveals the presence of *hlyA* (haemolysin A), *hlyIII* (haemolysin III), and *hlyC* (the haemolysin transport protein). These are virulence factors related to the invasiveness and toxicity of the bacteria, and are absent in the genomes of the commensal strains (Chajęcka-Wierzchowska et al. 2017).

None of the commensal strains harbour vancomycin-resistance genes; by contrast, most pathogenic strains have five, thus belonging to the VRE group and, consequently, being of high epidemiologic relevance (Centers for Disease Control and Prevention 2019). The development of multidrug-resistant strains from those initially innocuous has been exacerbated in the past 50 years, and this behaviour is strongly related to human activity (Beceiro et al. 2013). Some of the commensal strains studied contain the innate betalactam, aminoglycoside and macrolide resistances expected in the genus (Table 5) (Freitas et al. 2021) and they also harbour resistance genes to less common antibiotics such as fluoroquinolones and elfamycins. To successfully deal with antibiotics, not only resistance genes are important, but also antibiotic efflux pumps. Genes encoding these membrane proteins were found in all ten strains; however, it is remarkable that Aus0085 is not only resistant to a broader range of antibiotic categories (nucleosides and tetracyclines) but also contains additional efflux pumps that are absent in their counterparts. The inherent antibiotic resistance may result from natural selective pressure to adapt to a particular niche and does not necessarily reflect human activity. *Enterococcus* does not exclusively inhabit the animal gut but is also found in plants, sand, and soil (Dapkevicius et al. 2021). These habitats also harbour *Streptomyces,* an actinobacterium from which many widely used antibiotics are produced, such as kanamycin, streptomycin, erythromycin, tetracyclin, and even vancomycin (Quinn et al. 2020). This microorganism uses these molecules as defence mechanisms and has developed intrinsic resistance to them, which can be transferred to other microorganisms via horizontal gene transfer (Seipke et al. 2012). Thus, a primitive *Enterococcus* strain may have acquired these resistance genes by interacting with *Streptomyces*; over time, the strains that acquired these genes may have been favoured by the environment to survive, creating the intrinsically resistant genus we know today. The pool of antibiotic resistance genes in the environment that can be horizontally transferred between organisms is called the environmental resistome and is distributed in both commensal and pathogenic microorganisms (Wright 2010). Furthermore, it is not just bacteria that contribute to the environmental resistome, as mobile resistance genes also occur in environmental bacteriophages, making them part of this phenomenon (Das et al. 2022). To note, this study found that the plasmids belonging to Aus0085, DO, V1164 and V1836 contain antibiotic resistance genes (*lsa, ermB, aph-D, ant(6’), lsaE, sat, catA*), while the plasmids from commensal strains do not possess any resistance genes. It is worth mentioning that strains V1164 and V1836 harbour all of their vancomycin resistance genes in their plasmids, while Aus0004 and Aus0085 carry them in their chromosome.

#### Enterococcal metabolic features relevant to food fermentation and preservation

On the other hand, QD-2 shares 248 accessory elements with the other commensal strains. Of these, those related to carbohydrate metabolism constitute the predominant category, with transport-system genes for PTS sugars, such as mannitol, ascorbate, sorbitol, and glucitol/sorbitol. PTS is a phosphotransferase system that facilitates the metabolism of many monosaccharides, disaccharides, and other carbohydrate derivatives that depend on phosphoenolpyruvate. For microorganisms growing in dairy products such as cheese, possessing the necessary means to internalise sugars using PTS is of utmost importance (Flórez and Mayo 2015). In dairy products, lactose is the main carbon source for *Enterococcus*; however, this disaccharide is not the only carbohydrate that this microorganism can use. This was demonstrated with the dbCAN3 tool, which found several starch, sucrose, xylan, α/β-glycan, trehalose, pectin, chitin, rhamnose, and arabinan hydrolases in the QD-2 genome. Chitin is the second most abundant polysaccharide in nature, after cellulose. The ability to degrade cellulose has been previously described in gut enterococcal strains, and their chitin-degrading capacity has been shown to inhibit the growth of *Fusarium solani*, a phytopathogenic fungus (Atwa et al. 2022; Kohl et al. 2022). In the context of the artisanal Cotija cheese, *E. faecium* QD-2 might be capable of using yeast debris as a carbon source, as these are part of the cheese microbial community (Escobar-Zepeda et al. 2016).

Another set of important elements associated with cheese ripening found in the commensal strains is the proteolytic system. LAB are microorganisms that require an external source of certain amino acids, as they are auxotrophic to many of them (Dapkevicius et al. 2021). To meet their amino acid requirements, these dairy microorganisms break down and metabolise casein from the medium to grow and develop (Martino et al. 2018). This is useful not only for these microorganisms but also for the manufacturers of fermented food products, as proteolysis is one of the main sources of flavours and textures in fermented food (Dapkevicius et al. 2021). Previously, we characterised the proteolysis system in *E. faecium* D, it contains a large variety of genes encoding proteins that break down (Clp), internalise (Opp, Dpp, Dpt), and further metabolise casein from the medium (Pep) (Olvera-García et al. 2018). In the present study, we observed that strain QD-2 possesses almost all the genes encoding the proteins previously described, in addition to many components absent in strain D, such as *clpQ, clpC,* and *pepP* (Table S1)*.* The generation of bitter flavours could be controlled by PepP, an aminopeptidase that tightly controls the production and breaking down of Xaa-Pro-Xaa peptides, which are responsible for producing bitter flavours in cheese (Savijoki et al. 2006).

Another flavour-related metabolic feature found in food-enterococcal strains is the lipolytic system, producing compounds such as esters and free fatty acids, which give fermented foods their characteristic flavour and aroma (Martino et al. 2018). Many compounds derived from lipolysis and fatty acid metabolism, such as 2-heptanone, 2-nonanone, butanoic, hexanoic, and octanoic acids, are fragrant (Vélez et al. 2023). Our previous study showed that *E. faecium* D contains genes related to lipolysis, such as a lipase, two acetylesterases, and a carboxylesterase (Olvera-García et al. 2018). QD-2 genome annotation through KAAS reveals the presence of genes encoding one tributyrin esterase (*estA*), two acetylesterases (*aes*), and two carboxylesterases (*yvaK*, QD2_01579). It has been described that tributyrin esterase, EstA, is a key protein in the development of desirable aromas in cheese, alongside the aminopeptidase PepP previously described (Engels et al. 2022).

Other aroma-associated molecules produced by fermentation are volatile compounds such as ethanol, acetate, acetone, diacetyl, acetoin, and 2, 3-butanediol (Sarantinopoulos et al. 2002; Martino et al. 2018). Analysis of the QD-2 genome unveils the presence of genes encoding enzymes related to the production of acetoin (*ilvBIG*, *alsCD*, *budA*), ethanol, acetaldehyde, and acetate (*adhEC*). Furthermore, it was also found that QD-2 possesses the citrate operon *citCDEFGX*. Many aromas found in cheese, such as acetate, may also be derived from citrate metabolism (Sarantinopoulos et al. 2002).

Besides desirable compounds, some molecules produced during food fermentation are biogenic amines, such as putrescin, tyramine, histamine, and cadaverine, which are products of the decarboxylation process of ornithine, tyrosine, histidine, and lysin, respectively (Olvera-García et al. 2018). The undesirable presence of these compounds in food is commonly associated with health disorders for the consumer, such as diarrhoea, headache, vomiting, and tachycardia, and could also have a direct effect on carcinogenesis. The presence of biogenic amines is usually an indicator of food quality (Wójcik et al. 2020). We previously showed that although *E. faecium* D contains the genes necessary to produce tyramine, putrescin, and ornithine, these compounds could not be detected at significant levels in Cotija cheese via HPLC (Olvera-García et al. 2018). The analysis of the *E. faecium* QD-2 genome reveals the presence of *tyrCD, aguA*, and *argF*, associated with the synthesis of the biogenic amines mentioned above. The presence of these genes in the genome of QD-2 may not correlate with the significant production of biogenic amines in the same way as in strain D.

Bacteriocin-associated elements were shared between the commensal strains, corresponding to enterocin B (*entB*), a bacteriocin-immunity protein, and ABC transporters, except strain IQ110 which lacks *entB*. Enterocin B found in strains D, QD-2 and ATCC 8459 was identical, sharing 78% identity with enterocin B from QD-2 and 53% with carnobacteriocin A from *Carnobacterium maltaromaticum*. Enterocin B is an antimicrobial peptide belonging to the IIa bacteriocin group that possesses substantial activity against many food-related pathogens, i.e., *Listeria monocytogenes* (Ankaiah et al. 2018). The evolutionary history of *Enterococcus* suggests that the genus emerged as a ramification of *Carnobacterium*, which, in turn, evolved from a *Vagococcus*-like ancestor (Dapkevicius et al. 2021). An ancestral carnobacteriocin A-like molecule was probably encoded in the common ancestor of *Enterococcus* and *Carnobacterium*; later, as evolution took place, this primitive bacteriocin diverged into enterocin B and carnobacteriocin A since both are very similar (Casaus et al. 1997). This divergence still prevails, as certain variants of enterocin B only share 60% identity with other enterocin B variants (Qiao et al. 2020).

### Bacteriocin expression in *Enterococcus faecium* QD-2

*Enterococcus* is one of the most diverse genera in bacteriocin production, as these peptides are active against a wide variety of phyla, being of special interest given the current antibiotic resistance crisis (Almeida-Santos et al. 2021). Based on the whole-genome sequencing of QD-2 and its analysis on BAGEL4, eight bacteriocins were predicted in its genome, whose ORFs correspond to 615 and 616 (predicted bacteriocins), 621 (enterocin A, *entA*, Wu et al. 2022), 718 (*uviB-*like, Dupuy et al. 2004), 901 and 902 (enterocin X, *enxA/enxB,* Hu et al. 2010), 2142 (enterolysin A, Nilsen et al. 2003), and 2596 (enterocin B, *entB*, Casaus et al. 1997). It is worth noting that the activity of all but three (ORFs 615, 616, and 718) have been previously characterised. However, sequences 615 and 616 were identified as putative bacteriocins by O’Keeffe et al. (1999) (herein ORF1 and ORF2, respectively). More recently, our team identified enterocin 29 (Ent29α), a bacteriocin produced by an enterococcal strain found in a fermented sausage, which shares 100% identity with ORF 616 after its signal peptide has been excised and contains a consensus sequence related to antilisterial activity (Escamilla-Martínez et al. 2017). The putative bacteriocins 615 and 616 contain the signal peptide cleavage sequence GG, present in class II bacteriocins (Nes et al. 2013).

Enterocin A is particularly relevant for being the first widely studied class-IIa bacteriocin of enterococcal origin, especially for its activity against the foodborne pathogen *L. monocytogenes* (Wu et al. 2022). Enterocin B is a bacteriocin with activity against many pathogens such as *Acinetobacter baumanii, Staphylococcus aureus,* and *L. monocytogenes*; it also inhibits the formation of biofilms and possesses activity against human cancer cell lines (Ankaiah et al. 2018; Wu et al. 2022). Additionally, enterocin B and enterocin A can act synergically with one another, enhancing their antimicrobial activity (Casaus et al. 1997). Similarly, enterocin X is a class-IIb bacteriocin formed by two synergic peptides (Xα and Χβ) and can be found in *E. faecium* strains that produce enterocin A and B. It has been reported that ORFs 901 and 902 share the GXXXG motifs usually found in enterocin X (Hu et al. 2010).

*E. faecium* QD-2 has shown antilisterial activity in agar diffusion tests. Based on the available information about the biochemical characterization of enterocins A and X (ORFs 621, 901, and 902, respectively), the present study focused on ORFs 615 and 616 (Mendoza 2019). We assessed the transcription probability of these ORFs through an *in-silico* analysis of their promoter sequences and contrasted it against ORFs 901 and 902. iPro70-FMWin finds patterns by comparing canonical sigma 70 promoter sequences, considers the distance between regions -10 and -35, and calculates the probability of a functional promoter; thus, a score close to 1 represents a highly probable promoter sequence (Rahman et al. 2018). Two highly probable promoter sequences were located upstream from ORFs 615 and 901, with scores of 0.7860/0.7860 and 0.9981/0.9981 for regions -10/-35, respectively, while ORFs 616 and 902 showed lower scores, 0.0914/0.0914 and 0.4973/0.1140, respectively. These results suggest that these ORFs could be organised as bicistronic operons, which makes sense for enterocin X, described as a class-IIb two-peptide bacteriocin (Wu et al. 2022).

**Fig. 3:**
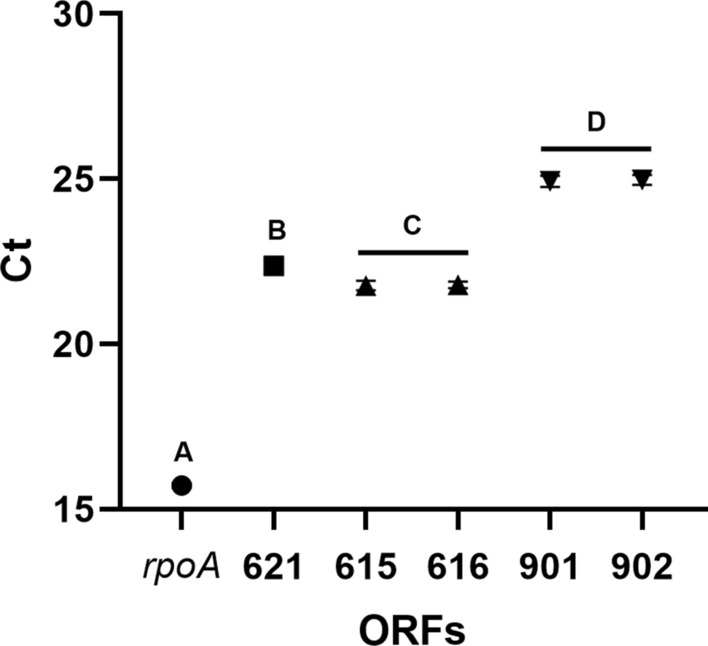
Ct values for all transcripts. Different letters represent a significant difference. ($\alpha$< 0.05)

The transcription rate of ORFs 615, 616, 901, and 902 was evaluated through an RT-qPCR. The housekeeping gene *rpoA,* which encodes the α-subunit of RNA polymerase, was selected as the normalization gene because its expression is fairly constant. It has proven reliable in RT-qPCR procedures in *Clostridium perfringens* and *Campylobacter jejuni* (Williams and Ghanem 2022; Ritz et al. 2009). The expression of ORF 621 was also considered as a baseline. All Ct values obtained from our analysis yielded a variance ratio lower than 1%, suggesting that the experimental procedure was reproducible and the results are reliable (Fig. 3).

**Fig. 4:**
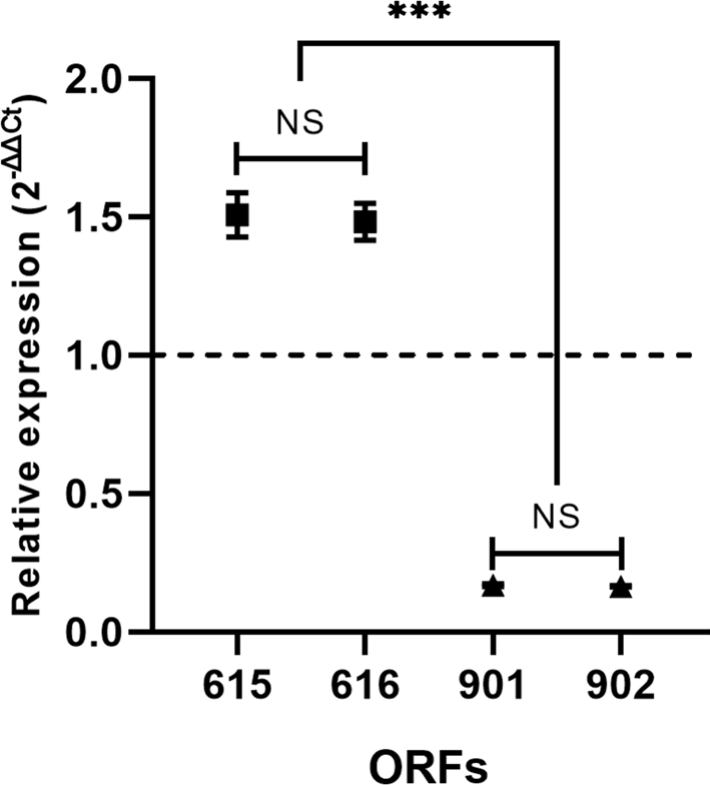
Relative expression of ORFs 615, 616, 901, and 902 compared to 621 (dotted line). Different groups represent a significant difference (NS = *p* > 0.05, *** = *p* < 0.05)

Normalised ΔCt values of ORFs 615, 616, 901, and 902 were compared against the ΔCt value for enterocin A (ORF 621) to calculate their relative expression, ΔΔCt. (Fig. 4). A clear difference between the expression of 615–616 and 901–902 relative to 621 can be observed, but there is no significant difference between the expression of 615 and 616, nor between 901 and 902. A one-way ANOVA and a *post-hoc* Tukey test found significant differences between all genes, except within 615–616 (*p* = 0.9995) and 901–902 (*p* = 0.9999). This behaviour, coupled with the strength of the promoters found upstream of these ORFs, suggests that putative bacteriocins 615–616 and 901–902 are co-expressed. This is also consistent with the genomic proximity between them. The expression of operons 615–616 and 901–902 is 1.5 times higher and 0.8 times lower, respectively, compared to enterocin A.

## Conclusions

The current discussion on the probiotic and biotechnological applications of enterococci, as well as on clinical concerns, is still a widely debated topic. We have observed that enterococci harbour many virulence and resistance factors in their genome, whether from hospital or food-related environments. However, the specific factors contained in and expressed by a given strain are highly dependent on the environment, as not all niches share the same stress conditions. Pathogenic strains contain more genes related to multidrug resistance, colonization ability, and host toxicity, whereas food-related enterococcal strains tend to carry genes related to flavour, aroma, texture development, carbohydrate uptake, lipolysis, and bacteriocins. Particularly, the *E. faecium* QD-2 strain expresses bacteriocins A and X, in addition to two putative additional ones encoded by ORFs 615 and 616, which need to be further characterised. The difference between strains from different environments is clear evidence of the influence of human activities on these organisms, as they are essential components not only of our own microbiota but also of the food we eat and the places we inhabit.

## Supplementary Information

Below is the link to the electronic supplementary material.

Supplementary file1 (DOCX 17 KB)

## Data Availability

The datasets generated during and/or analysed during the current study are available in the Genbank repository under accession number CP130822.
