# Supplementary material for: Study of an Enterococcus faecium strain isolated from an artisanal Mexican cheese, whole-genome sequencing, comparative genomics, and bacteriocin expression
Source: Antonie Van Leeuwenhoek. 2024 Feb 23;117(1):40. doi: 10.1007/s10482-024-01938-0 (PMC10891205; doi:10.1007/s10482-024-01938-0)
Supplement: Supplementary file 1 — Supplementary file1 (DOCX 17 KB) [file 10482_2024_1938_MOESM1_ESM.docx]

**Table S1.** Proteolysis systems present in *Enterococcus faecium* QD-2 and *E. faecium* D (Supplementary)

| **Proteolytic system** | **Gene** | **Activity** | **QD-2^A^** | **D^B^** |
| --- | --- | --- | --- | --- |
| Clp complex | *clpP* | ATP-dependant Clp protease | 2 | 1 |
|  | *clpE* | ATP-dependant Clp protease | 1 | 1 |
|  | *clpB* | ATP-dependant Clp protease | 1 | 2 |
|  | *clpL* | ATP-dependant Clp protease | 1 | 1 |
|  | *clpX* | ATP-dependant Clp protease | 1 | 1 |
|  | *clpC* | ATP-dependant Clp protease | 1 | - |
|  | *clpQ* | ATP-dependant Clp protease | 1 | - |
| ABC-mediated oligopeptide transport | *oppA* | Extracellular solute-binding protein | 1 | 1 |
|  | *oppB* | Protein-dependant transport system, inner membrane component | 1 | 2 |
|  | *oppC* | Oligopeptide transport permease, transmembrane domain | 1 | 1 |
|  | *oppD* | Oligo/dipeptide ABC transporter | 1 | 2 |
|  | *oppF* | Oligo/dipeptide ABC transporter | 1 | 2 |
| Dipeptide and tripeptide transport | *dppC* | Dipeptide transport permease | 1 | 1 |
|  | *dppD* | ATP-binding dipeptide transport protein | 2 | - |
|  | *dppF* | ATP-binding dipeptide transport protein | 2 | - |
|  | *dppE* | ATP-binding dipeptide transport protein | 1 | 1 |
|  | *dtpT* | Di/tripeptide transporter | 1 | 1 |
| Endopeptidases | *pepO* | Metallopeptidase | 1 | 1 |
|  | *pepF* | Oligoendopeptidase | 1 | 1 |
| Dipeptidases | *pepV* | Zinc-dependant aminopeptidase | 2 | 2 |
| Aminopeptidases | *pepC* | Aminopeptidase | 1 | 1 |
|  | *pepA* | Glutamyl aminopeptidase | 2 | 1 |
|  | *pcp* | Pyroglutamyl-peptidase | 1 | - |
| Proline peptidases | *pepP* | Proline aminopeptidase | 2 | - |
|  | *pepQ* | Proline aminopeptidase | - | 1 |
| Other | *pepT* | Tripeptide aminopeptidase | 1 | 1 |
|  | *pepB* | Oligoendopeptidase | - | 1 |
|  | *pepS* | Aminopeptidase | 1 | 1 |

A: This study; B: **Olvera-García et al. (2018)**
